# Intracellular mediators of transforming growth factor β superfamily signaling localize to endosomes in chicken embryo and mouse lenses in vivo

**DOI:** 10.1186/1471-2121-8-25

**Published:** 2007-06-25

**Authors:** Ramya Rajagopal, Shunsuke Ishii, David C Beebe

**Affiliations:** 1 Dept. Ophthalmology and Visual Sciences, USA; 2 Dept. Cell Biology and Physiology, Washington University, Saint Louis, Missouri, USA; 3 Laboratory of Molecular Genetics, RIKEN Tsukuba Institute, Ibaraki, Japan

## Abstract

**Background:**

Endocytosis is a key regulator of growth factor signaling pathways. Recent studies showed that the localization to endosomes of intracellular mediators of growth factor signaling may be required for their function. Although there is substantial evidence linking endocytosis and growth factor signaling in cultured cells, there has been little study of the endosomal localization of signaling components in intact tissues or organs.

**Results:**

Proteins that are downstream of the transforming growth factor-β superfamily signaling pathway were found on endosomes in chicken embryo and postnatal mouse lenses, which depend on signaling by members of the TGFβ superfamily for their normal development. Phosphorylated Smad1 (pSmad1), pSmad2, Smad4, Smad7, the transcriptional repressors c-Ski and TGIF and the adapter molecules Smad anchor for receptor activation (SARA) and C184M, localized to EEA-1- and Rab5-positive vesicles in chicken embryo and/or postnatal mouse lenses. pSmad1 and pSmad2 also localized to Rab7-positive late endosomes. Smad7 was found associated with endosomes, but not caveolae. *Bmpr1a *conditional knock-out lenses showed decreased nuclear and endosomal localization of pSmad1. Many of the effectors in this pathway were distributed differently in vivo from their reported distribution in cultured cells.

**Conclusion:**

Based on the findings reported here and data from other signaling systems, we suggest that the localization of activated intracellular mediators of the transforming growth factor-β superfamily to endosomes is important for the regulation of growth factor signaling.

## Background

Cells often respond to stimuli through cell surface receptors. In response to their specific ligands, receptor molecules generate distinct biological response(s) by activating cytoplasmic signaling molecules. These effectors often reach the nucleus and alter gene regulation. Precise regulation of signaling is required to provide an appropriate response. Many reports suggest that endocytosis is an important mechanism for regulating signaling by cell surface receptors. Endocytosis may target ligand-receptor complexes to lysosomes for degradation [1,2] or recycle receptors to the plasma membrane [3,4], thereby regulating the number of receptors that are available for ligand binding.

Recent studies indicate that endocytosis also promotes and, in some cases, may be necessary for the transmission of signals from receptors to the nucleus. Endosomes appear to promote signaling by serving as scaffolds for the activated components of signal transduction pathways. For example, nerve growth factor (NGF) activates persistent signaling by the small GTPase, Rap1 on endosomes in PC12 cells. Activated NGF receptor (TrkA), mitogen-activated protein kinase (MAPK), the guanine nucleotide exchange factor, C3G, and the adaptor molecules, CrkL, Shp2 and Gab2 all co-immunoprecipitate with activated Rap1. Disruption of the endosomal compartment with brefeldin A inhibits the activation of Rap1 by NGF [5]. Subsequent studies demonstrated an important role for endosome-bound signaling complexes in the transmission of NGF-mediated survival signals from axon terminals to the cell body in vivo [6]. Loss of dynamin, a protein that is required for the formation of endocytic vesicles, results in the formation of an excess of neural cells during *Drosophila *neurogenesis, a phenotype similar to that resulting from disruption of Notch signaling. Ligand-dependent activation of Notch signaling requires the function of dynamin [7]. Similarly, endosome formation is required for Wnt signaling [8]. The ability of epidermal growth factor (EGF) to activate MAPK is prevented by the over expression of a dominant negative form of dynamin, which inhibits the formation of endocytic vesicles [9]. Signaling by activated platelet-derived growth factor (PDGF) receptors occurs on endosomes [10] and the activation of MAPK by fibroblast growth factor receptors requires receptor internalization [11]. Therefore, 'signaling endosomes' are increasingly recognized as important components in the transmission of signals from activated receptors to the nucleus [12].

The formation of endosomes is also required in the transforming growth factor-β (TGF-β) signaling pathway. Effector Smad proteins transduce TGF-β signaling by linking the serine-threonine kinase activity of TGF-β receptors to changes in gene expression in the nucleus. Endocytosis of the TGF-β receptor is required for the TGF-β-induced nuclear translocation of Smad2 and its subsequent signaling in HeLa cells [13] and in cultured human mesangial cells [14]. Also, distinct endocytic pathways are required for TGF-β receptor signaling in Mv1Lu and HEK293T cells [15].

**Figure 1:**
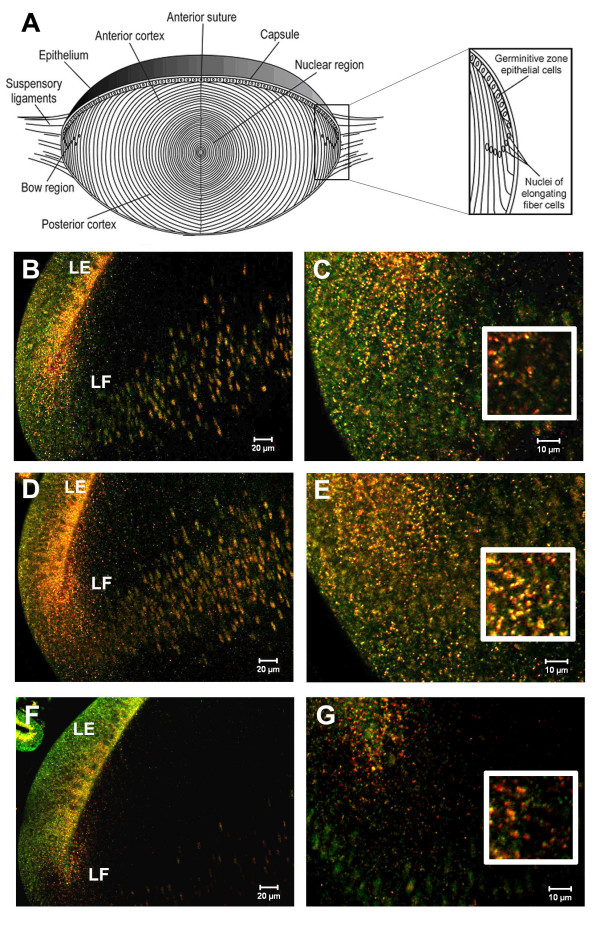
Smad4, Smad7 and SARA co-localize on small cytoplasmic vesicles with the early endosomal marker EEA1 in E7 chicken lenses. A. Diagram of the organization of the lens. B. EEA1 (green), Smad4 (red). D. EEA1 (green), Smad7 (red). F. EEA1 (green), SARA (red). C, E and G are higher magnification images of the regions outlined in figures B, D and F respectively. The insets in C, E and G show 2X magnified images of antibody stained cytoplasmic vesicles (LE – lens epithelia, LF – lens fibers)

Bone morphogenetic proteins (BMPs), which are members of the transforming growth factor-β (TGF-β) superfamily, regulate several aspects of lens development. Vertebrate lens formation commences when the neuroectoderm of the optic vesicle comes in close contact with the head ectoderm, resulting in the formation of an ectodermal thickening called the lens placode. The lens placode invaginates and eventually separates from the surface ectoderm to give rise to the lens vesicle. Targeted deletion of BMP4 or 7 in mice results in the failure of lens placode formation and the absence of a lens [16-18]. Cells in the posterior of the lens vesicle stop dividing and elongate to form lens fiber cells. Over expression of a dominant negative form of the type I BMP receptor, *Bmpr1b *(*Alk6*) inhibits lens fiber cell formation in mouse embryo lenses [19] and the BMP antagonist, noggin, interferes with lens fiber cell differentiation in both chicken and mouse embryos [19,20]. The lens is then comprised of an anterior layer of epithelial cells, which maintain the ability to proliferate, and a posterior mass of post-mitotic fiber cells. The lens grows by the proliferation of equatorial epithelial cells, which subsequently differentiate into peripheral fiber cells (Figure 1A). Targeted deletion from the lens of the gene encoding the type1 BMP receptor, *Bmpr1a (Alk3) *results in lenses that are smaller in size, with thinner lens epithelia. The fiber cells in these lenses fail to fully withdraw from the cell cycle and later swell and degenerate [21].

**Figure 2:**
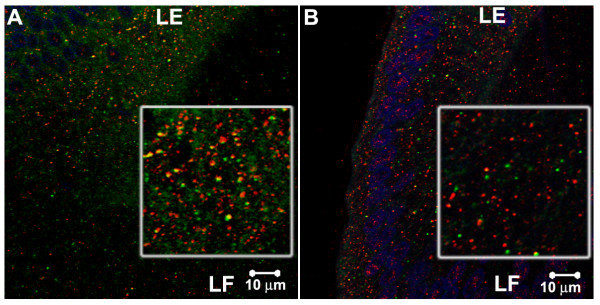
Smad7 co-localizes with early endosomal marker, EEA1, on cytoplasmic vesicles, but not to an appreciable degree with Caveolin-1, a marker for caveolae, in E7 chicken lenses. A. Smad7 (red), EEA1 (green) and TOPRO (blue). B. Smad7 (red), Caveolin-1 (green) and TOPRO (blue). The insets show 2X magnified images of antibody stained cytoplasmic vesicles, more clearly demonstrating co-localization (yellow) for Smad7 and EEA1, but much less so for Smad7 and Caveolin-1.

In this report, we show that intracellular molecules that mediate signaling by the TGF-β superfamily localize on endosomes in vivo in chicken embryo and postnatal mouse lens cells. These effectors include receptor-activated Smads (R-Smads), the co-Smad, Smad4, the inhibitory Smad (I-Smad), Smad7 and scaffold proteins and transcriptional repressors in the Smad pathway. All were found in association with early (EEA-1- and/or Rab5B-positive) endosomes. We also found that that pSmad1 and pSmad2 localized on a small number of Rab7-positive late endosomes. In lenses that lacked the type1 BMP receptor, *Bmpr1a*, levels of the activated R-Smad, pSmad1, were greatly decreased on endosomes and in the nucleus. We noted several differences between the distribution of these components in cells in vitro and in vivo and suggest that the endosomal localization of these molecules has functional significance.

## Results

### Several components of the TGFβ signaling pathway localize to early endosomes

**Figure 3:**
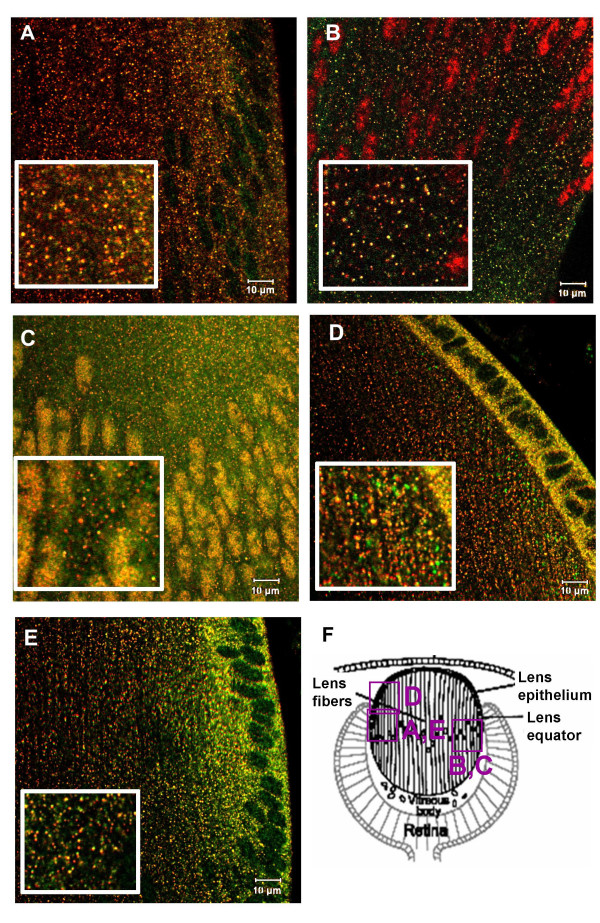
Endosomal localization of pSmad1, pSmad2, TGIF, C184M and c-Ski in P3 mouse lenses. A. Rab5B (red), pSmad1 (green). B. EEA1 (green), pSmad2 (red). C. EEA1 (green), TGIF (red). D. EEA1 (green), C184M (red). E. c-Ski (green), C184M (red). F is a diagram of the neonatal mouse lens showing the regions that are represented in each of the images.

In previous studies from our laboratory, we identified cytoplasmic vesicles in chicken embryo lens cells that stained with antibodies to pSmad1 or pSmad2 [20]. We later showed that these pSmad1 and pSmad2-positive vesicles co-localize with Rab5b and EEA1, markers for early endosomes, in chicken embryo and mouse lens cells [21] (Figure 3A and 3B). Visualization of these vesicles was facilitated by using a confocal microscope to view antibody staining in thick, detergent-permeabilized tissues slices. We extended these studies by determining whether other proteins that are known to serve as downstream components of TGFβ superfamily signaling were found in association with endosomes.

Antibodies against SARA, Smad4 and Smad7 stained abundant cytoplasmic vesicles in chicken embryo lens epithelial and fiber cells (Figure 1 B-G). Many of these vesicles also labeled with antibodies to the early endosomal marker EEA1, suggesting that SARA, Smad4 and Smad7 associate with early endosomes in the lens cell cytoplasm. Smad4 is the common-mediator Smad, which forms hetero-oligomers with activated R-Smads. This complex then translocates to the nucleus and, together with co-activators or repressors, regulates transcription [22]. Although the endosomal localization of TGF-β receptors has been shown to be important for signaling [13,14], Smad4 has not previously been localized to endosomes. Smad7 negatively regulates TGF-β signaling by targeting the TGF-β receptor for degradation by recruiting Smurf2, an E3 ubiquitin ligase [22,23]. In cultured cells, Smad7 has been reported to localize on Caveolin-1-positive membrane compartments, which are distinct from endosomes [15,24]. However, we found that Smad7 and EEA1 co-localize on chicken embryo lens sections (Figures 1D and 1E, 2A). Double labeling with antibodies against Smad7 and Caveolin-1 revealed no appreciable co-localization (Figure 2B). Hence, our results suggest that Smad7 localizes to endosomes to a greater extent than to caveolae and, therefore, may function differently in lens cells in vivo than in tissue culture cells. SARA is a FYVE-domain adapter protein that recruits Smad2 to the TGFβ receptor and is required for the phosphorylation of Smad2 by the activated TGFβ receptor [25]. SARA has been shown to localize on endosomes in cultured cells [14,15,26]. Our studies show that this also occurs in vivo.

We next examined the distribution of less well-studied components of TGFβ/BMP signaling. C184M is a cytoplasmic protein that binds to c-Ski, a transcriptional co-repressor of TGF-β signaling. When over expressed, C184M inhibits the nuclear translocation of Smad2 [27]. Antibodies to C184M stained vesicular structures that co-stained with EEA1 in mouse lens cells (Figure 3D). Since C184M was reported to bind to c-Ski, we confirmed that c-Ski localized to many of the same vesicles as C184M (Figure 3E). Thus, in lens cells, C184M and c-Ski are endosome-associated proteins. In a similar manner, TGIF, another transcriptional co-repressor of TGFβ signaling, was found associated with EEA1-positive endosomes (Figure 3C).

**Figure 4:**
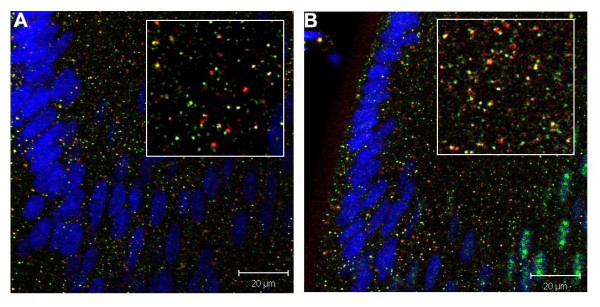
Localization of pSmad1 and pSmad2 on late endosomes in P3 mouse lens cells. A. Rab7 (red), pSmad1 (green), TOTO-1 (blue). B. Rab7 (red), pSmad2 (green), TOTO-1 (blue). Both pSmad1 and pSmad2 co-localize with Rab7 on a small number of cytoplasmic vesicles. Nuclear staining for pSmad1 and pSmad2 is mostly obscured by the strong fluorescence of the nucleic acid stain, TOTO-1.

To analyze whether activated Smads localized to endocytic compartments other than early endosomes in mouse lens cells, we performed double-labeling with antibodies to pSmad1 or pSmad2 and Rab7, a marker for late endosomes. (Figure 4A and 4B). Counts of several regions in representative sections showed that 15–20% of vesicles that stained for pSmad1 or pSmad2 were also were Rab7-positive.

**Figure 5:**
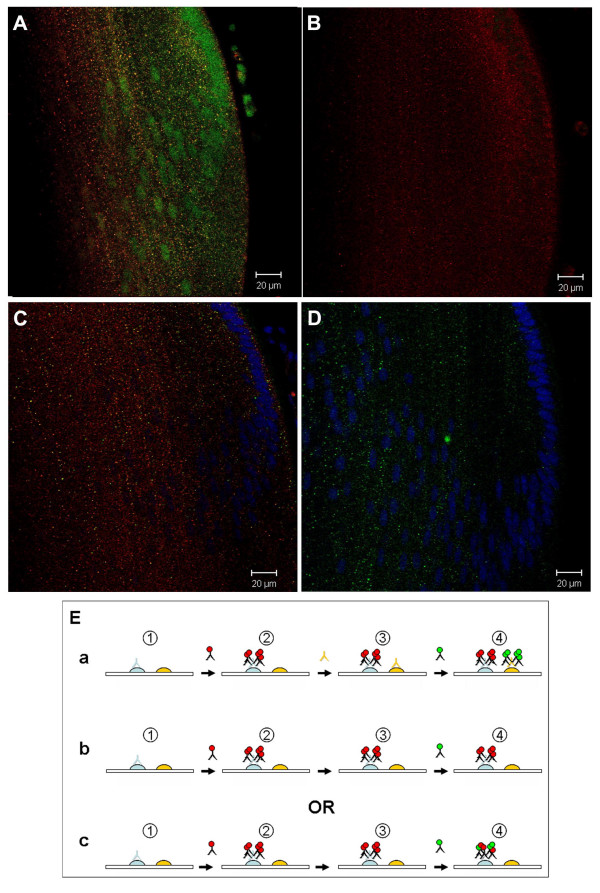
Double immunostaining of lens sections with two rabbit primary antibodies (A, C) and controls in which the second primary antibody was omitted (B, D). A. Rab5B (red), pSmad1 (green). B. Rab5B (red), no pSmad1 primary antibody. No green staining from the second anti-rabbit secondary antibody was detected. C. Rab7 (green), Rab5B (red), TOTO-1 (blue). D. Rab7 (green), no Rab5B primary antibody, TOTO-1 (blue). No red staining from the second anti-rabbit secondary antibody was detected. E. Diagram illustrating the method used for double labeling (**a**) and the alternative outcomes of the control studies for specificity (**b, c**). In **a**, the first rabbit primary antibody (light blue, 1) binds to its antigen and is localized by a fluorescent-labeled anti-rabbit secondary antibody (red, 2). The tissue is washed, fixed in formalin and washed again. The second rabbit primary antibody is then added (yellow), which binds to the second antigen (3). The second anti-rabbit secondary antibody (green) is added and binds to the second rabbit primary antibody (4). The sequence shown in **b **and **c **illustrates the controls used in the present study. The first two steps are as in **a**, but the second rabbit primary antibody is omitted (3), providing no antibody for the second anti-rabbit fluorescent antibody to bind (green, 4). In **b**, the second fluorescent-labeled anti-rabbit secondary antibody (green) does not bind the first primary antibody (blue) and the section remains singly labeled. In the steps shown in **c**, the second fluorescent-labeled anti-rabbit secondary antibody (green) binds to the first rabbit primary antibody (blue), resulting in spurious "co-localization." Since staining by the second anti-rabbit secondary antibody was not seen, as shown in B and D, **b **accurately reflects the results obtained in the present studies; the events shown in **c **were not observed.

**Figure 6:**
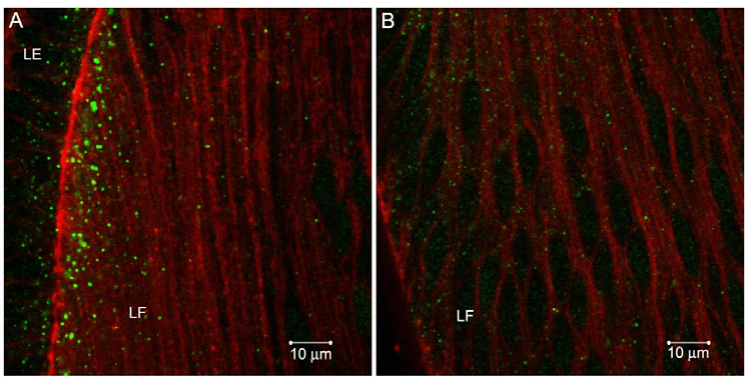
Double staining of lens sections with antibody against EEA1 (green) and phalloidin (red) in A and antibody to EEA1 (green) and antibody to β-tubulin (red) in B. No co-localization of either filamentous actin or tubulin with EEA1 was detected (LE- lens epithelium, LF- lens fibers).

In each case, the use of a single antibody gave results that closely resembled those obtained in our double-labeling studies. However, our conclusions depend on the co-localization of antibodies to two proteins on the same vesicles. In many cases, the two primary antibodies used in our double-labeling studies were produced in rabbits. To co-localize two primary antibodies produced in the same species, we developed a double-labeling protocol that avoided the cross-labeling of the primary antibodies by the anti-rabbit secondary antibodies. This method involved incubating with saturating levels of secondary antibody and post-fixing the thick sections after staining with the first set of primary and secondary antibody. These treatments prevented the second fluorescent-labeled, anti-rabbit secondary antibody from binding to the first primary antibody. To assure the specificity of this double-label method, we analyzed control samples for each pair of primary antibodies used in this study. Examples of such controls are illustrated in Figure 5A–D. Control sections were not exposed to the second primary antibody, but were sequentially incubated with each of the secondary antibodies, as illustrated in Figure 5E. These sections invariably did not show detectable staining by the second anti-rabbit secondary antibody. Furthermore, in all of our studies, the two antibodies used did not co-label all vesicles. If there were cross-binding of the second anti-rabbit secondary antibody to the first primary antibody, all vesicles that were labeled by the first antibody would also have been labeled by the second. Furthermore, not all molecules tested co-localized with endosomal markers. For example, staining for the cytoskeletal proteins F-actin and β-tubulin showed that they did not localize on EEA1-positive structures (Figure 6A and 6B). These tests show that the method used for double labeling provided an accurate assessment of the co-localization of the two antigens being studied.

**Figure 7:**
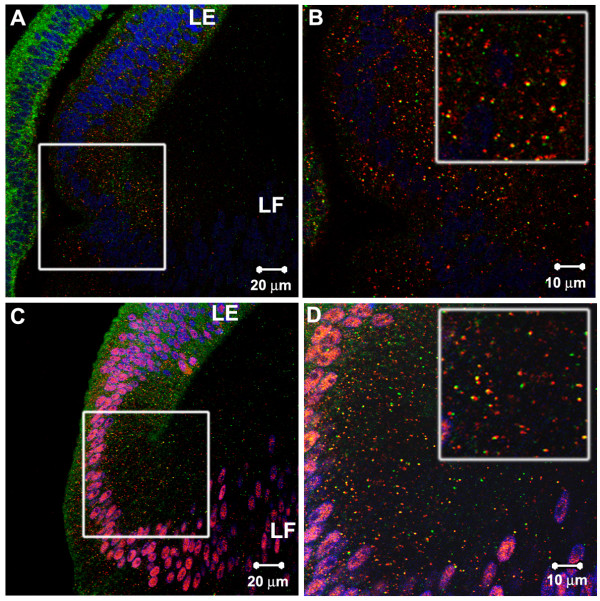
Localization of Smad4 and pSmad1/5/8 on early endosomes in E7 chicken lenses. A. EEA1 (green), Smad4 (red) and TOPRO (blue). C. EEA1 (green), pSmad1/5/8 (red) and TOPRO (blue). B and D are higher magnification images of the regions outlined in figures A and C respectively. The insets in B and D show 2X higher magnification images of antibody-stained cytoplasmic vesicles. Both Smad4 and pSmad1/5/8 show co-localization (yellow) with EEA1.

As a final test for the specificity of our methods, we double-labeled for Smad4 and EEA1 or pSmad1/5/8 and EEA1 using primary antibodies raised in different species (Fig. 7A–D). This approach showed co-localization of Smad4 and EEA1 (Figures 7A and 7B) and pSmad1/5/8 and EEA1 (Figures 7C and 7D) on cytoplasmic vesicles, similar to our observations using our double-labeling protocol with rabbit antibodies. This experiment confirmed that co-localization of two antigens using antibodies raised in the same species did not result from cross-binding of the secondary antibodies.

### Differential localization of components of the TGFβ signaling pathway in chicken embryo lens epithelial and fiber cells

**Figure 8:**
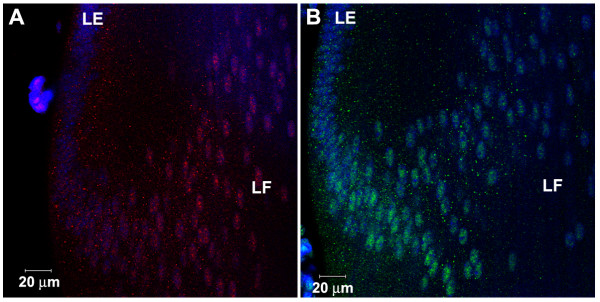
Localization of pSmad1 (A) and pSmad2 (B) in lens epithelial (LE) and fiber cells (LF). There is obvious nuclear localization of pSmad1 and pSmad2 in the fiber cells, where as the nuclear localization is hardly discernible in the epithelial cells. Punctate staining is abundant in the cytoplasm of epithelial and fiber cells. Nuclei are labeled using TOPRO (blue).

**Figure 9:**
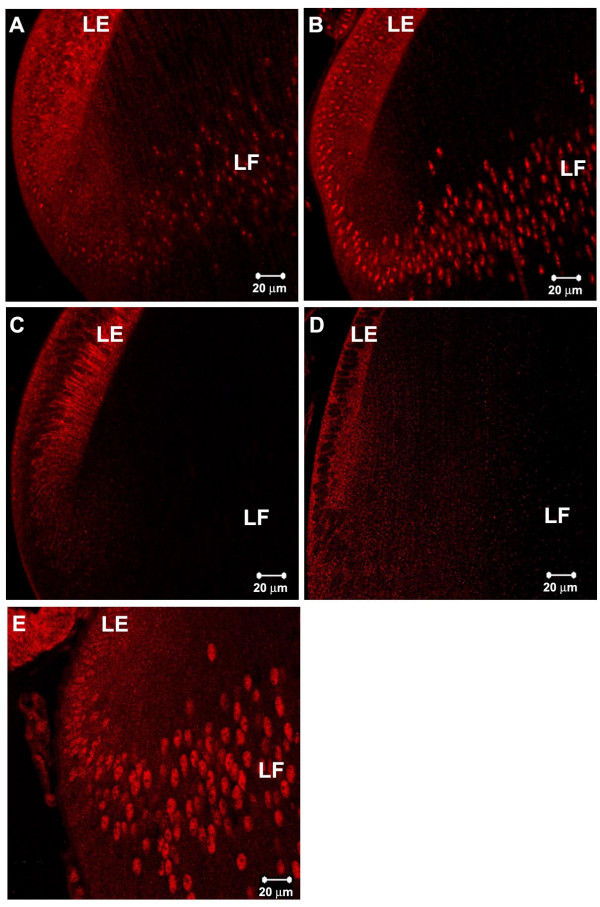
Localization of Smad4 (A), Smad7 (B),) and Smad6 (C), c-Ski (D) and TGIF (E) in lens epithelial (LE) and fiber cells (LF). Nuclear localization of Smad4 and TGIF is seen mainly in lens fiber cells. Smad7 is seen in the nuclei of both epithelial and fiber cells, although staining is stronger in fiber cell nuclei. Both I-Smads, Smad7 and Smad6, are abundant in the cytoplasm of lens epithelial cells. c-Ski is found mainly to the cytoplasm. A-C are sections of E7 chicken lenses and D-E are P3 mouse lenses.

As in our previous studies, antibodies to pSmad1 and pSmad2 stained the nuclei of elongating lens fiber cells, but did not stain to an appreciable degree the nuclei of lens epithelial cells (Figure 8A and 8B). We saw a similar distribution using antibodies to Smad4 (Figure 9A), Smad7 (Figure 9B), Smad6 (Figure 9C), c-Ski (Figure 9D) and TGIF (Figure 9E); abundant staining of endosomes in epithelial cells, but lower staining or no staining in epithelial cell nuclei. In contrast, both endosomes and the nuclei of fiber cells were stained with antibodies to these proteins. Antibodies to the cytoplasmic scaffold protein SARA and to C184M labeled abundant cytoplasmic vesicles in epithelial and fiber cells, but did not stain the nuclei of either cell type. These results show that, in lens epithelial cells, activated signaling complexes containing phosphorylated R-Smads, Smad7, Smad4 and the transcriptional repressors c-Ski and TGIF are present on endosomes, but, unlike in fiber cells, conditions in epithelial cells do not promote the accumulation of these proteins in nuclei. These results suggest that there are mechanisms that regulate the selective nuclear accumulation of activated R-Smads and other mediators in vivo. These mechanisms have not yet been revealed using in vitro studies, where activated pSmads usually translocate directly to the nucleus.

### Reduction in BMP signaling decreases the endosomal and nuclear localization of R-Smads

**Figure 10:**
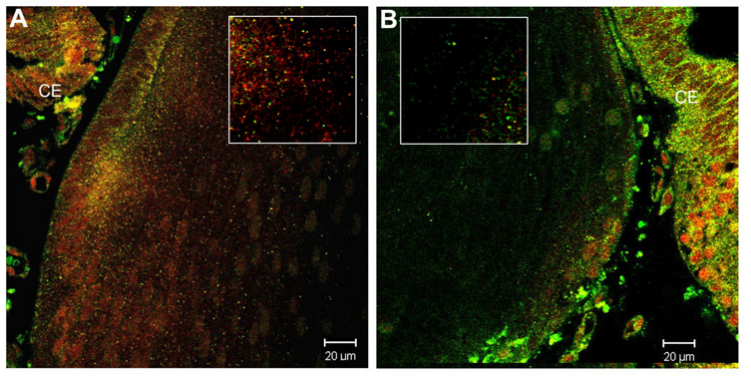
Marked reduction in the endosomal and nuclear localization of pSmad1 in *Bmpr1a*^*CKO *^lenses. A. WT (Cre-negative) lens; Rab5B (green), pSmad1 (red). B. *Bmpr1a*^*CKO *^(Cre-positive) lens Rab5B (green), pSmad1 (red). Both the number of endosomes and the relative number of Smad1-positive endosomes is reduced in the *Bmpr1a*^*CKO *^lens. In B, note the relative difference in staining intensity for pSmad1 and Rab5b in the ciliary epithelium (CE), compared to the *Bmpr1a*^*CKO *^lens.

To investigate whether the endosomal localization of activated R-Smads in lens epithelial and fiber cells occurred in response to BMP receptor signaling, we stained with antibodies to pSmad1 in lenses lacking the type1 BMP receptor, *Bmpr1a *(*Alk3*) [21]. *Bmpr1a *conditional knock-out (*Bmpr1a *^*CKO*^) lenses had substantially lower levels of pSmad1 in the nuclei of lens fiber cells, compared to lenses that expressed *Bmpr1a*. Epithelial and fiber cells from these lenses also contained many fewer endosomes and a smaller number of these stained for pSmad1 than in wild type lenses (Figure 10A and 10B). In the *Bmpr1a *^*CKO *^mice, Cre recombinase is not expressed in the ciliary epithelium, a tissue that is adjacent to the lens and which depends on BMP7 for its normal development [28]. Therefore, we used the ciliary epithelium as an internal reference for the levels of pSmad1 staining. Unlike the reduced amount of nuclear and endosomal pSmad1 in lens cells, the ciliary epithelium had normal, high levels of nuclear and endosomal pSmad1. Removing *Bmpr1a *from the lens cells did not eliminate pSmad1 staining. Since all three type1 BMP receptors, *Acvr1 *(*Alk2*), *Bmpr1a *(*Alk3*) and *Bmpr1b *(*Alk6*) are expressed in the lens [19,29], it is possible that the low level of pSmad1 remaining in *Bmpr1a *^*CKO *^lenses was due to phosphorylation of Smad1 by signaling through Acvr1 and/or Bmpr1b receptors.

## Discussion

We found that positive regulators of TGFβ superfamily signaling, including the activated R-Smads, pSmad1 and pSmad2, the co-Smad, Smad4, the cytoplasmic scaffold proteins, SARA and C184M, and the negative regulators of Smad signaling, Smad7, TGIF, and c-Ski, localized on early endosomes in vivo. Activated R-Smads were also present on late endosomes. When BMP signaling was reduced, the level of pSmad1 on endosomes and in the fiber cell nuclei decreased.

The localization on endosomes of activated R-Smads, co-Smad and negative regulators of Smad-dependent transcription, such as TGIF and c-Ski are novel findings. These, together with the co-localization of c-Ski and its binding protein, C184M on cytoplasmic vesicles, suggests that endosomes act as platforms for the assembly of both positive and negative components of the Smad signaling pathway. Understanding the functional significance of these observations will require methods that have not yet been widely applied to cells in vivo.

Previous studies localized TGFβ superfamily signaling in vivo by detecting the distribution of pSmad1 and pSmad2 in chicken and Xenopus embryos [30,31]. These studies used histochemical detection in paraffin-embedded tissue sections. Although this is a sensitive method, it may obscure the endosomal localization of pSmads and other mediators of TGFβ superfamily signaling. Thus, while these authors showed abundant pSmad staining in the cytoplasm of all tissues examined, this staining was not obviously localized to vesicular structures. Using confocal microscopy to view thick, detergent-permeabilized tissue sections that had not been embedded in paraffin demonstrated that pSmads and other mediators of TGFβ superfamily signaling localized to punctate cytoplasmic structures, along with EEA1 and Rab5B or Rab7.

### Differences between results of the present in vivo and previous in vitro studies

The results reported in this study contrast in many ways to those obtained from the study of cultured cells treated with ligands of the TGFβ superfamily. For example, activated Smads are rarely described in the cytoplasm of cultured cells, although we found abundant endosome-associated Smads in vivo. In fact, in lens epithelial cells, activated Smads were easily seen on endosomes, but did not accumulate to appreciable levels in nuclei. These differences in localization may be cell type specific, as studies of the endosomal localization of signaling components have not previously been performed on lens cells. They may also be related to the different manner in which cells are typically exposed to growth factors in vivo and in vitro.

Smad7 has previously been found associated with cytoplasmic membranes, but these vesicles were caveolin-1-positive compartments (caveolae), not endosomes [15,24]. These authors concluded that the pathway for the Smad7-dependent degradation of TGFβ receptors (via caveolae) is distinct from the pathway by which these receptors activate downstream components (via endosomes). When we assessed the relative distribution of Smad7 on endosomes and caveolae, our results showed that Smad7 associates to an appreciable degree with endosomes in vivo, but not with caveolae. It is possible that Smad7 is regulated via a different pathway in lens cells. Another study showed strong co-localization of the polyoma virus VP1 protein with EEA1 and of EEA1 with Caveolin-1 in mouse fibroblasts. This observation suggests that Caveolin-positive vesicles carrying the virus fuse with EEA1-positive early endosomes [32]. This observation raises the possibility that these pathways overlap in some cell types.

We are aware of no studies describing the localization of negative transcriptional regulators of TGFβ signaling, like c-Ski and TGIF, to endosomes. Similarly, the association of Smad4 with activated R-Smads has been assumed to occur in the cytoplasm, since Smad4 has not previously been detected on endosomes. Finally, although C184M was previously found exclusively in the cytoplasm, it was not shown to associate there with endosomes [27]. Some of these differences may be due to the imaging methods used in the present study. For instance, cytoplasmic staining for pSmad1 and 2 has been shown previously in embryonic tissues, but it was not evident that this staining was associated with vesicles (see [28,30] for examples).

The differences between our work and previous in vitro studies may be due to the methods that have been classically used to study growth factor signaling in cultured cells. In vitro, cells are often first 'starved' for growth factors and then exposed to levels sufficient to saturate all cell surface receptors. Following this acute exposure, signaling intermediates rapidly translocate to the nucleus. In vivo, especially during development and other non-traumatic events, cells are likely to be exposed to ligand levels that increase gradually over minutes or hours, as a growth factor is synthesized and diffuses to its target. After cells are exposed to a stimulus, they are likely to activate feedback mechanisms to modulate their response to stimulation. This may account for the presence of Smad7 on endosomes in vivo, but not in cultured cells. In vitro studies suggest that phosphorylated R-Smads move rapidly from the receptor to the nucleus and do not reside in the cytoplasm for an appreciable time. Our observations show that, in vivo, a substantial fraction of the total pSmad1 and pSmad2 is, at any time, associated with endosomes in the cytoplasm.

At the time they were removed from the eye, the lens cells studied in the present work had been chronically exposed to BMPs and other members of the TGFβ superfamily for days [16,17,19-21]. Therefore, the localization of signaling components and complexes is likely to reflect their steady-state distribution in the cells. Our results suggest that this steady state is characterized by the endosomal association of active R-Smads, I-Smad, co-Smad and Smad effector molecules. Most of these proteins are thought to have their primary function in the nucleus. Since at steady state, the distribution of molecules within different cell compartments reflects the amount of time they spend in these compartments, our observations suggest that components of the TGFβ signaling pathway spend a substantial proportion of their time on endosomes. Understanding the functions of these endosome-associated complexes may provide a more complete picture of Smad signaling and its regulation.

In vitro studies permit the analysis of signaling pathways using sophisticated analytical methods, most of which are not yet practical for in vivo studies. Conversely, in vivo studies reveal aspects of signaling that may not be appreciated using cultured cells. The current study identified several aspects of TGFβ superfamily signaling in vivo that are not typical of what has been seen for in vitro studies. These warrant further study to determine whether they are due to differences in cell type, differences between cells in vivo and in vitro, or some of both.

### Differential localization of TGFβ signaling components to the cytoplasm and nucleus of lens epithelial and fiber cells

Lens epithelial cells showed high levels of all Smad signaling components, including pSmads, in their cytoplasm, but not their nuclei. This observation suggests that there are factors that regulate whether activated Smads primarily localize to the cytoplasm or the nucleus. Smad distribution could be regulated by altering the relative rates of nuclear import and export of activated R-Smads [33,34]. Alternatively, recent studies showed that Sno-N (*ski*-related novel gene), a transcriptional repressor that is related to c-Ski, suppresses TGFβ signaling by sequestering Smads in the cytoplasm [35]. Their cytoplasmic location in vivo raises the possibility that c-Ski and C184M may play a similar role in regulating the distribution of Smads between the cytoplasm and nucleus. Our studies detected the I-Smad, Smad6, in the epithelial cell cytoplasm, but not in the fiber cells. Smad6 may, therefore, prevent the nuclear localization of Smad signaling complexes in lens epithelial cells. Finally, lens epithelial and fiber cells abut different ocular compartments, with epithelial cells exposed to aqueous humor and fiber cells to vitreous humor. Different amounts of TGFβ family members or other growth factors in these compartments might account for the differences seen in the subcellular localization of activated Smads and other TGFβ signaling components in epithelial and fiber cells.

An example of the potential complexity of cytoplasmic signaling is provided by MAPK signaling in Drosophila eye development. MAPK is activated (phosphorylated) early in eye development, but is held in the cytoplasm. When the cells are later exposed to BMP and hedgehog ligands, the activated MAPK translocates to the nucleus, where it regulates development [36]. Although the mechanisms that regulate the subcellular distribution of activated signaling molecules are not yet well understood, in Drosophila or vertebrates, we suggest that such mechanisms function downstream of TGFβ superfamily receptors in the lens in vivo.

## Conclusion

Several mediators of TGFβ superfamily signaling localize to endosomes in chicken embryo and mouse lens cells. These include adapter molecules and positive and negative regulators of TGFβ signaling. We suggest that, in typical in vitro studies, the acute exposure of cultured cells to high concentration of ligand may not reveal all aspects of TGFβ superfamily signaling. Activated R-Smads were differentially localized in lens epithelial and fiber cells, suggesting that there are as yet unidentified mechanisms that regulate the nuclear accumulation of these molecules. Reduction of BMP signaling by targeted deletion of *Bmpr1a *decreased the endosomal and nuclear localization of pSmad1, a finding that is consistent with a functional role for endosome-associated signaling complexes in vivo. Innovative approaches will be needed to delineate the functions of these endosome-associated complexes in intact tissues.

## Methods

### Materials

Antibody to phosphorylated Smad1 (pSmad1) was obtained from Upstate Biotechnology, anti-pSmad1/5/8, anti-pSmad2 and anti-β-tubulin were from Cell Signaling Technology (Danvers, MA), anti-Smad4, Smad6, Smad7, SARA, c-Ski, Rab5B and Rab7 were from Santa Cruz Biotechnology (Santa Cruz, CA), anti-EEA1 was from Calbiochem (San Diego, CA) and the mouse antibodies against EEA1 and Caveolin-1 were from BD Transduction Laboratories (San Diego, CA). The C184M antibody was described previously [27]. Alexa-Fluor-labeled secondary antibodies, Alexa-Fluor-labeled phalloidin, TOTO-1 and TOPRO were obtained from Molecular Probes (Eugene, OR). VectaShield was from Vector labs (Burlingame, CA).

### Animals

Fertile chicken embryos were obtained from CBT Farm (Chestertown, Maryland) and were incubated at 38°C until the embryos reached seven days of embryonic development (E7). Mice lacking *Bmpr1a (Alk3) *in the lens were generated by mating *Bmpr1a *floxed mice [37] with LeCre mice, which express Cre recombinase in the lens [38], as described previously [21]. Cre-negative, homozygous floxed lenses were considered as wild type. Postnatal day 3 (P3) mouse eyes were dissected, fixed and sectioned.

### Immunohistochemistry

Chicken embryo lenses dissected from the eye and isolated mouse eyes were fixed in 10% formalin for 1–2 hours, embedded in 4% agar and sliced in 100 μm thick sections using a vibrating tissue slicer (EM Sciences, Hatfield, PA). The sections were permeabilized and blocked in PBS supplemented with 0.5% Triton X-100 and 5% goat serum and labeled with antibodies specific for phosphorylated Smad1 (pSmad1), pSmad2, Smad4, SARA, Smad6, Smad7, TGIF, c-Ski, C184M or β-tubulin. Sections were usually double-labeled with markers for early endosomes (EEA1 or Rab5B) or a marker for late endosomes (Rab7). Optimal dilutions of primary antibodies were determined. Anti-mouse or rabbit secondary antibodies and Alexa-Fluor labeled phalloidin were used at 1:1000 dilution. Antibody staining was detected using a Zeiss 510 confocal microscope.

For double-labeling with two primary antibodies raised in the same species, sections were first incubated in one primary antibody overnight, extensively washed in PBS containing 0.5% Tween-20 for 1 hour in Netwells (EM Sciences) [39] and incubated in the first secondary antibody overnight. The sections were washed again in PBS with 0.5% Tween-20 for an hour and fixed in 10% formalin for 2 hours. This was followed by washes with PBS and incubation in the second primary antibody overnight. The sections were again washed in Netwells and incubated in the second secondary antibody for 2 hours, washed again in PBS with Tween 20 and mounted using a 1:1 dilution of VectaShield in PBS. Control sections for the double-labeling experiments omitted the second primary antibody. This strategy would reveal any non-specific staining arising due to binding of the second secondary antibody to the first primary antibody.

## Authors' contributions

RR carried out the immunohistochemistry experiments using wild-type and *Bmpr1a*^*CKO *^mouse lenses and chicken embryo lenses. DCB designed and supervised the study and carried out immunohistochemistry experiments using chicken embryos lenses. RR and DCB wrote the manuscript. SI provided an essential reagent (C184M antibody) for the studies and helped draft the manuscript. All authors read and approved the final manuscript.
